# Assessment of resistance profiles and virulence genes in *Escherichia coli* from fresh vegetables: implications for food safety and consumer health in Ogun State, Nigeria

**DOI:** 10.1186/s13213-026-01864-8

**Published:** 2026-07-14

**Authors:** Itohan Ebunoluwa Abatan, Lenis Saweda O. Liverpool-Tasie, Taofik Akinyemi Shittu, Adebukunola Mobolaji Omemu, Muinah Adenike Fowora, Oluwakemi Abosede Ojo, Oluwasola Abayomi Adelusi, Adewale Olusegun Obadina

**Affiliations:** 1 Department of Food Science and Technology, Federal University of Agriculture, Abeokuta, Nigeria; 2 Department of Agricultural, Food, and Resource Economics, Michigan State University, Michigan, USA; 3 Department of Hospitality and Tourism, Federal University of Agriculture, Abeokuta, Nigeria; 4 Department of Molecular Biology and Biotechnology, Nigerian Institute of Medical Research, Lagos, Nigeria; 5 Department of Biotechnology and Food Technology, University of Johannesburg, Johannesburg, South Africa

**Keywords:** Vegetables, *Escherichia coli*, AMR, Virulence genes, Food safety, Consumer health

## Abstract

**Purpose:**

Fresh vegetables are essential human dietary components but are increasingly implicated in foodborne infections caused by *Escherichia coli*. Contamination may occur during cultivation, irrigation, handling, and retail display, and the presence of pathogenic or antimicrobial-resistant *E. coli* poses significant public health risks. Despite widespread vegetable consumption in Ogun State, Nigeria, limited data exist on the resistance and virulence characteristics of *E. coli* in these foods. This study therefore assessed antimicrobial resistance profiles and virulence genes in *E. coli* isolated from tomatoes, carrots, and cucumbers sold across three senatorial districts of Ogun State.

**Method:**

A cross-sectional survey was conducted between March and October 2023, yielding 509 vegetable samples collected aseptically during three visits. Samples were enriched, cultured on MacConkey agar, and identified using standard microbiological methods. Thereafter, antimicrobial susceptibility testing was performed using the Kirby–Bauer disc diffusion method. Genomic DNA was extracted from the microbe, followed by Polymerase Chain Reaction (PCR) to detect key virulence (*stx1, stx2, eae*) and resistance (*blaSHV, blaTEM, blaCTX, NDM, IMP, KPC, OXA-48*) genes.

**Results:**

Of the 509 vegetables analysed, 73 (14.4%) tested positive for *E. coli*: 34 (14.4%) from tomatoes, 27 (24.1%) from carrots, and 12 (7.5%) from cucumbers. Carrots from Ogun Central had an *E. coli* contamination rate of 9.8% (11/112), while cucumbers from Ogun East had a contamination rate of 1.3% (2/160). Among 34 *E. coli* isolates confirmed and recovered from the vegetables, 12 originated from Ogun Central, 9 from Ogun East, and 13 from Ogun West. Isolates showed resistance to all antibiotics except gentamicin and levofloxacin. Virulence genes *stx1* and *stx2* were detected in 3 and 2 isolates, respectively. Also, 47% of isolates carried resistance genes, and 15% carried virulence genes.

**Conclusion:**

Fresh vegetables across the three senatorial districts of Ogun State were contaminated with *E. coli*, with 15% of isolates carrying virulence genes and 47% carrying antimicrobial resistance genes. The low detection of virulence genes indicates relatively low pathogenic potential, as higher levels typically signal greater infection risk. However, the presence of both virulence and resistance genes still highlights important public health concerns. These findings emphasize the need for improved farming hygiene, stricter antimicrobial regulation, and stronger surveillance.

## Introduction

Antibiotics were once regarded as a panacea for all human infections, yet their efficacy proved fleeting, with contemporary microorganisms evolving resistance to nearly all available antimicrobials (Selvarajan et al. 2022). Antimicrobial resistance (AMR) is the evolution of microorganisms to express increased or complete resistance to antimicrobial agents that were previously effective in eliminating them during an infection (Nelson et al. 2019). AMR can spread through various pathways, including contact with animal or human carriers, environmental reservoirs such as contaminated water and soil, and the food chain (Blaak et al. 2014). Fresh produce, particularly vegetables consumed raw, has emerged as an important vehicle for the transmission of antimicrobial-resistant bacteria due to contamination during cultivation (e.g., irrigation with wastewater, use of manure), post-harvest handling, and retail display in traditional markets. Such contamination may facilitate the transfer of resistant bacteria to consumers, contributing to the community spread of AMR (Kläui et al. 2024). Traditional food markets are present globally, offering affordable food to millions of consumers. In low- and middle-income countries, these markets play a crucial role as primary sources of household food and contribute significantly to local employment (FAO 2024). However, traditional markets face heightened food safety concerns due to rapid economic, demographic, and dietary changes, coupled with limited capacities for food safety management, improper infrastructure, such as clean water, and inadequate storage facilities (DeWaal et al. 2022).

Antibiotic-resistant bacteria typically carry specific resistance genes corresponding to the antibiotics they resist. *Escherichia coli* (*E. coli*), part of the Enterobacteriaceae family, is a key bacterium responsible for diarrheal diseases in humans via faecal-oral transmission and is often used as an indicator of faecal contamination in food production (Hinthong et al. 2024). In Nigeria, *Shiga toxin-producing Escherichia coli* (STEC) with key virulence genes have been reported in food products, highlighting the regional public-health relevance of pathogenic *E. coli*. For example, a study of fresh beef and locally processed ready-to-eat meat products in Lagos detected STEC serogroups in a large proportion of samples, with 25% of isolates carrying major virulence factors, including *stx1*, *stx2*, and *eae* (intimin), and many isolates possessing combinations of two or more virulence genes (Fayemi et al. 2021). These pathogenic strains carry specific virulence genes essential for causing disease in humans and animals. The *eae* gene, responsible for producing intimin, is associated with EPEC, while the heat-labile toxin gene (lt) and heat-stable toxin gene (st) are linked to ETEC. The shiga toxin genes, *stx1* and *stx2*, are linked to STEC (Pakbin et al. 2021).

*E. coli* can serve as an important reservoir for antibiotic resistance genes, often carried on plasmids that can be transferred to neighboring bacteria through conjugation. In fresh produce environments, such as irrigation water, manure-amended soils, and farm surfaces, these plasmid-mediated genes can spread among environmental bacteria and plant-associated microbiota, creating a route for AMR transmission to vegetables (Tacão et al. 2014). ESBL-producing *E. coli* and other Enterobacteriaceae are particularly concerning, as they can persist in soil and irrigation water, exhibit co-resistance to multiple antibiotic classes, and potentially reach humans through contaminated produce, thus posing a significant public health risk (Castanheira et al. 2021). ESBL-producing bacteria typically carry specific resistance genes on plasmids, with common genes like *blaTEM*, *blaCMY-2*, and *blaOXA* found in bacteria of both human and animal origins (Bradford et al. 2001, Li et al. 2007). Infections caused by ESBL-producing bacteria tend to be more severe and can lead to prolonged hospital stays.

Carbapenem-resistant *E. coli* (CREC) represents another category of resistance. These gram-negative bacteria are resistant to carbapenems, a class of antibiotics typically reserved as the last line of defense against multidrug-resistant infections (Tompkins and van Duin 2021). CREC can cause serious infections such as intra-abdominal infections, pneumonia, urinary tract infections, and infections related to medical devices (Huang et al. 2024). Due to their high antibiotic resistance and frequent transmission between humans and animals, CREC infections are a significant public health and food safety concern. CREC strains often carry resistance genes for multiple classes of antibiotics, making treatment more challenging due to the presence of additional resistance mechanisms. Although reports of carbapenem-resistant *E. coli* (CREC) from fresh vegetables in Nigeria are limited, most available studies have focused on clinical isolates or animal sources with comparatively fewer investigations addressing carbapenem-resistant *E. coli* in fresh produce and irrigation water in West Africa. For example, CARBA-R *E. coli* were detected in vegetables and irrigation water in markets in Abidjan, Ivory Coast, and carbapenem-resistant *E. coli* pathovars were isolated from fresh-cut fruits in Accra, Ghana, demonstrating that carbapenem resistance can occur along the fresh produce food chain in the region (Ingrid et al. 2025; Nketiah et al. 2024). Furthermore, the combined assessment of phenotypic resistance, ESBL production, carbapenem resistance, and virulence gene carriage in vegetable-derived *E. coli* has not been sufficiently explored in traditional market settings. Understanding the general traits of ESBL *E. coli* and CREC strains in terms of antibiotic resistance and virulence is crucial for tracking and controlling their spread.

Evaluating AMR profiles, along with virulence and resistance genes in the food supply chain, is vital for ensuring the safety and quality of food, such as vegetables, from farm to table. The presence of pathogens in these vegetables can lead to resistant strains that are difficult to eliminate (Nelson et al. 2019). Despite their nutritional benefits, vegetables have been identified as carriers of antimicrobial-resistant pathogens, raising food safety concerns, particularly due to their frequent fresh consumption in raw form or with minimal processing (Olanbiwoninu et al. 2024). This study, therefore, aimed to examine the resistance profiles, virulence factors, and antimicrobial resistance genes of *E. coli* found in fresh vegetables from traditional markets in Ogun State, Nigeria.

## Materials and methods

### Sample collection

**Fig. 1:**
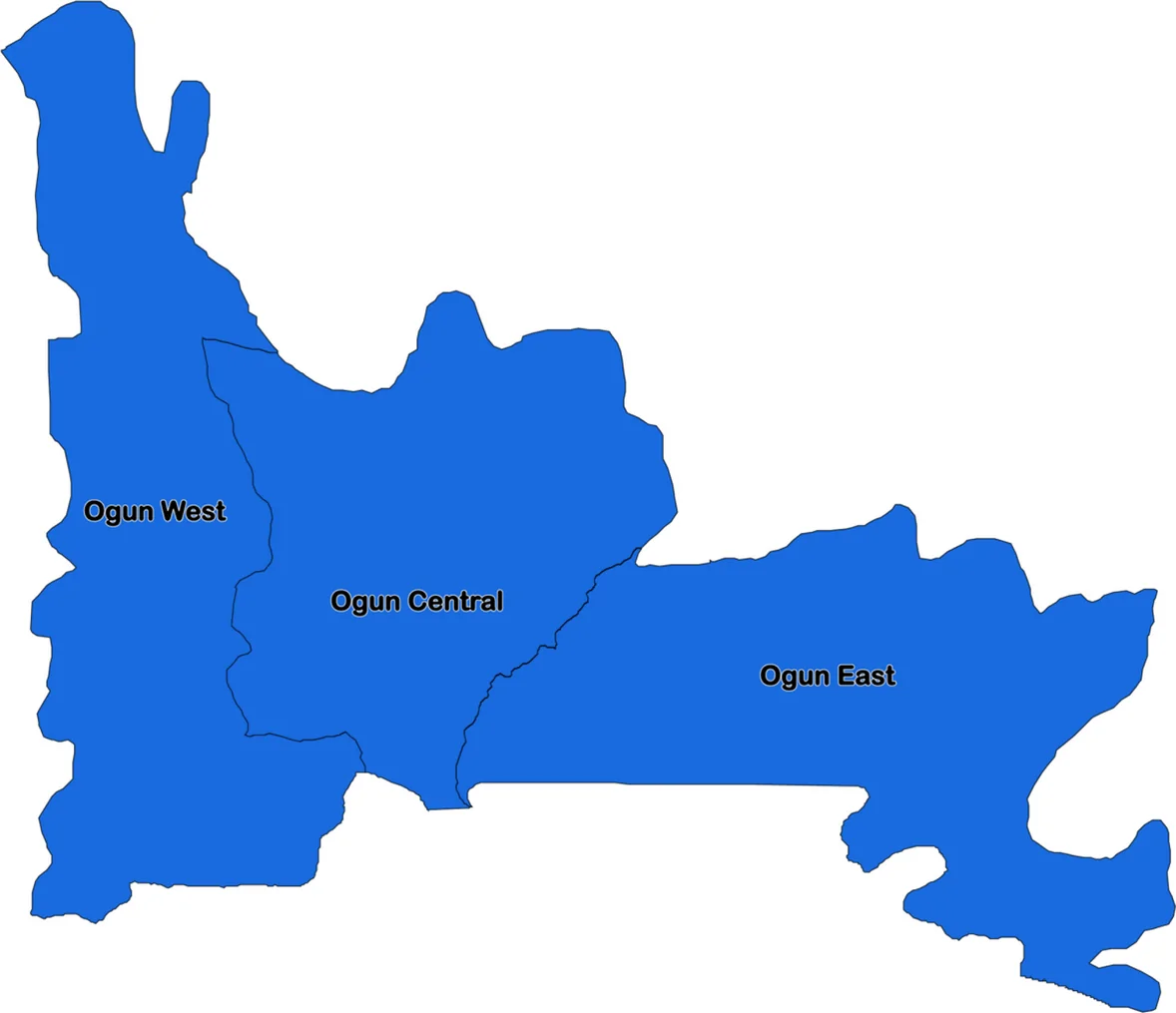
Map of Ogun State Showing Ogun West, Ogun Central, and Ogun East Senatorial Districts for Sample Collection

A total of 509 selected fresh vegetable samples were randomly collected in three visits from March 2023 to October 2023. The first visit was conducted in March (dry season), the second in May (early rainy season), and the third in October (late rainy season). During the first visit, a total of 174 fresh vegetable samples were collected, comprising tomato (n = 79), carrot (n = 41), and cucumber (n = 54). During the second visit, 172 fresh vegetable samples were obtained, including tomato (n = 79), carrot (n = 40), and cucumber (n = 53). In the third visit, 163 fresh vegetable samples were collected, consisting of tomato (n = 79), carrot (n = 31), and cucumber (n = 53). All samples were obtained from local traders across nine selected markets distributed within the three senatorial districts of Ogun State, Nigeria (Fig. 1). Each sample unit consisted of a single vegetable type obtained from an individual vendor per visit. Approximately 200—250 g of the edible portion was aseptically collected and treated as one analytical sample. Samples were processed independently to preserve variability across vendors and sampling periods. The vegetable samples were collected in sterile plastic bags, appropriately labelled, and transported in insulated cold storage (4—6 °C) to the laboratory for analysis. Microbiological processing commenced within 4—6 h of collection to minimize changes in microbial load prior to examination.

This study (MSU Study ID: STUDY00008064) was reviewed and approved by the Institutional Review Board of Michigan State University, the lead partner institution, with concurrence from Federal University of Agriculture, Abeokuta.

### Microbial isolation and identification

One (1) gram of sample was introduced into 9 ml of sterile peptone water in a prelabelled tube to allow physiological resuscitation and increase the sensitivity of relevant pathogen detection, and the tube was incubated at 37 ℃ for 24 h. A loopful of the inoculum was streaked onto MacConkey agar (MAC) plate and incubated aerobically at 37 °C for 24 h. Presumptive *Escherichia coli* isolates from a fresh MacConkey plate were sub-cultured by streaking onto Eosin Methylene Blue (EMB) agar plate and incubated aerobically at 37 °C for 24 h for purification*.* On EMB agar, *E. coli* typically produces dark colonies with a characteristic metallic green sheen due to strong acid production and dye precipitation (Sheth et al. 2022). Preliminary identification was based on colony morphology, gram staining and standard biochemical tests including indole production, methyl red, citrate utilization, triple sugar iron (TSI) and motiliy testing following established micorbiological procedures.

To improve identification accuracy, phenotypically presumptive isolates were further confirmed by polymerase chain reaction (PCR) targeting the specific gene of interest. Only isolates confirmed by molecular analysis were included in subsequent antimicrobial susceptibility testing.

### Antimicrobial susceptibility testing

The antimicrobial susceptibility test of isolates to antibiotics was determined on Mueller–Hinton agar by the disc diffusion method (Bauer et al. 1966). The antibiotics discs (Mast group, UK) used were: Gentamicin (10 µg), Azithromycin (15 µg), Amoxicillin-clavulanate (30 µg), Levofloxacin (5 µg), Cefuroxime (30 µg), Trimethoprim-sulfamethoxazole (25 µg), Ciprofloxacin (5 µg), Ceftriaxone (30 µg), Ampicillin (10 µg), Nitrofurantoin (300 µg), Imipenem (10 µg), Meropenem (10 µg), Cefoxitin (30 µg).

Plates were incubated aerobically at 35 ± 2 °C for 18–24 h. The diameters of inhibition zones were measured in millimetres using a digital calliper. Results were interpreted as susceptible, intermediate, or resistant according to the guidelines of the Clinical and Laboratory Standards Institute (Wayne 2010).

All susceptibility tests were performed in duplicate to ensure reproducibility. Quality control was performed using reference strain *Escherichia coli* ATCC 25922, and zone diameters were confirmed to fall within CLSI-recommended ranges.

### DNA extraction from isolates

Total genomic DNA was obtained from the identified pathogenic bacteria using the NIMR Kit (Lagos, Nigeria) following the manufacturer’s specifications.

### Polymerase Chain Reaction (PCR) Amplification of virulence determinant and resistance genes

The extracted DNA was subjected to PCR amplification to detect the virulence gene (*stx1*, *stx2* and *eae*) and resistance gene (*blaSHV*, *blaTEM*, *blaCTX*, *NDM*, *IMP*, *KPC,* and *Oxa-48*). PCR amplification was carried out using 25 µl of pre-mix (Accupower) containing taq (*Thermus aquaticus*) polymerase, dNTPs and MgCl_2_ and reaction buffer, 2 µL of template DNA, 1 µL each of forward and reverse primers (10 pmol/µL), and nuclease-free water to complete the final volume. Positive control strains previously confirmed to carry the respective target genes were included in each PCR run. A no-template control (NTC) was included in each PCR run, consisting of the complete reaction mixture with nuclease-free water substituted for template DNA, in order to monitor for reagent or environmental contamination. The primers, genes, and PCR conditions are as shown in Table 1. The amplified PCR products were analyzed using agar gel electrophoresis (1.5% agarose gel stained with 0.5 µg/mL ethidium bromide), and the gel was subsequently photographed. A 100 bp DNA ladder was used as a molecular size marker to determine amplicon sizes.

**Table 1:** Primers used in this study

Target	Genes	Primer sequence (5ʹ–3ʹ)	Product size (bp)	Conditions	References
Virulence genes	stx1 stx1	F:ACACTGGATGATCTCAGTGG R:CTGAATCCCCCTCCATTATG	614	35 cycles: 94 ℃ for 5 min, 94 ℃ for 30 s, 58 ℃ for 40 s, 72 ℃ for 45 s. Final extension: 72 ℃ for 10 min	Santaniello et al. 2006
	stx2 stx2	F:CCATGACAACGGACAGCAGTT R:CCTGTCAACTGAGCAGCACTTTG	779	35 cycles: 94 ℃ for 5 min, 94 ℃ for 30 s, 58 ℃ for 40 s, 72 ℃ for 45 s. Final extension: 72 ℃ for 10 min	El-Bastawisy et al. 2023
	eae eae	F:CCCGAATTGGCACAAGCATAAGC R:CCCGGATCCGTCTCGCCAGTATTCG	863	35 cycles: 95 ℃ for 5 min, 95 ℃ for 30 s, 55 ℃ for 30 s, 72 ℃ for 30 s. Final extension: 72 ℃ for 10 min	Schmidt et al. 1994
Resistance Genes	blaSHV blaSHV	F:CGCCTGTGTATTATCTCCCT R:CGAGTAGTCCACGAGAT CT	293	35 cycles: 95 ℃ for 5 min, 95 ℃ for 30 s, 50 ℃ for 30 s, 72 ℃ for 30 s. Final extension: 72 ℃ for 10 min	Bubpamala et al. 2018
	blaTEM blaTEM	F:TTTCGTGTCGCCCTTAT CC R:ATCGTTGTCAGAAGTAAGTTGG	409	35 cycles: 95 ℃ for 5 min, 95 ℃ for 30 s, 50 ℃ for 30 s, 72 ℃ for 30 s. Final extension: 72 ℃ for 10 min	Bali et al. 2010
	blaCTX blaCTX	F:CGCTGTTGTTAGGAAGTGTG R:GGCTGGGTGAAGTAAGTGAC	754	35 cycles: 95 ℃ for 5 min, 95 ℃ for 30 s, 50 ℃ for 30 s, 72 ℃ for 30 s. Final extension: 72 ℃ for 10 min	Ramachandran et al. 2017
	blaNDM blaNDM	F:GGGCAGTCGCTTCCAACGGT R:GTAGTGCTCAGTGTCGGC	439	35 cycles: 95 ℃ for 5 min, 95 ℃ for 30 s, 54 ℃ for 30 s, 72 ℃ for 30 s. Final extension: 72 ℃ for 10 min	Gurung et al. 2020
	KPC KPC	F:CATTCAAGGGCTTTCTTGCTGC R:ACGACGGCATAGTCATTTGC	340	35 cycles: 95 ℃ for 5 min, 95 ℃ for 30 s, 54 ℃ for 30 s, 72 ℃ for 30 s. Final extension: 72 ℃ for 10 min	Compain et al. 2014
	IMP IMP	F:TTGACACTCCATTTACDG R:GATYGAGAATTAAGCCACYCT	183	35 cycles: 95 ℃ for 5 min, 95 ℃ for 30 s, 54 ℃ for 30 s, 72 ℃ for 30 s. Final extension: 72 ℃ for 10 min	Ghasemian et al. 2019
	Oxa-48 Oxa-48	F:GCTTGATCGCCCTCGATT R:AAAGCCGGTGCCTCGTTTAG	281	30 cycles: 94 ℃ for 10 min, 94 ℃ for 40 s, 60 ℃ for 40 s, 72 ℃ for 1 min. Final extension: 72 ℃ for 7 min	Gurung et al. 2020

### Statistical analysis

Antibiotic resistance data were summarized using descriptive statistics and expressed as frequencies (n) and percentages (%). Differences in resistance frequencies among the tested antibiotics were evaluated using the Chi-square test. Statistical significance was set at *p* < 0.05. All analyses were performed using IBM SPSS Statistics version 30 (IBM Corp., Armonk, NY, USA).

## Results

### *E. coli* contamination of selected vegetable samples in all the senatorial districts

Table 2 shows the total number of vegetable samples collected during the present study. This included tomatoes (n = 237), carrots (n = 112), and cucumbers (n = 160). Of the 509 samples, a total of 73 (14.3%) tested positive for *E. coli*. A high contamination by *E. coli* was reported in carrots (24.1%), followed by tomatoes (14.4%) and cucumbers (7.5%).

**Table 2:** *E. coli* contamination of selected vegetables samples in all the senatorial districts

Vegetables	Total no of samples	Contaminated samples	No of positive *E. coli* samples
Tomatoes	237	88	34 (14.4%)
Carrot	112	39	27 (24.1%)
Cucumber	160	70	12 (7.5%)
Total	509	197	73 (14.4%)

### Overall prevalence of *E. coli* across senatorial districts

**Fig. 2:**
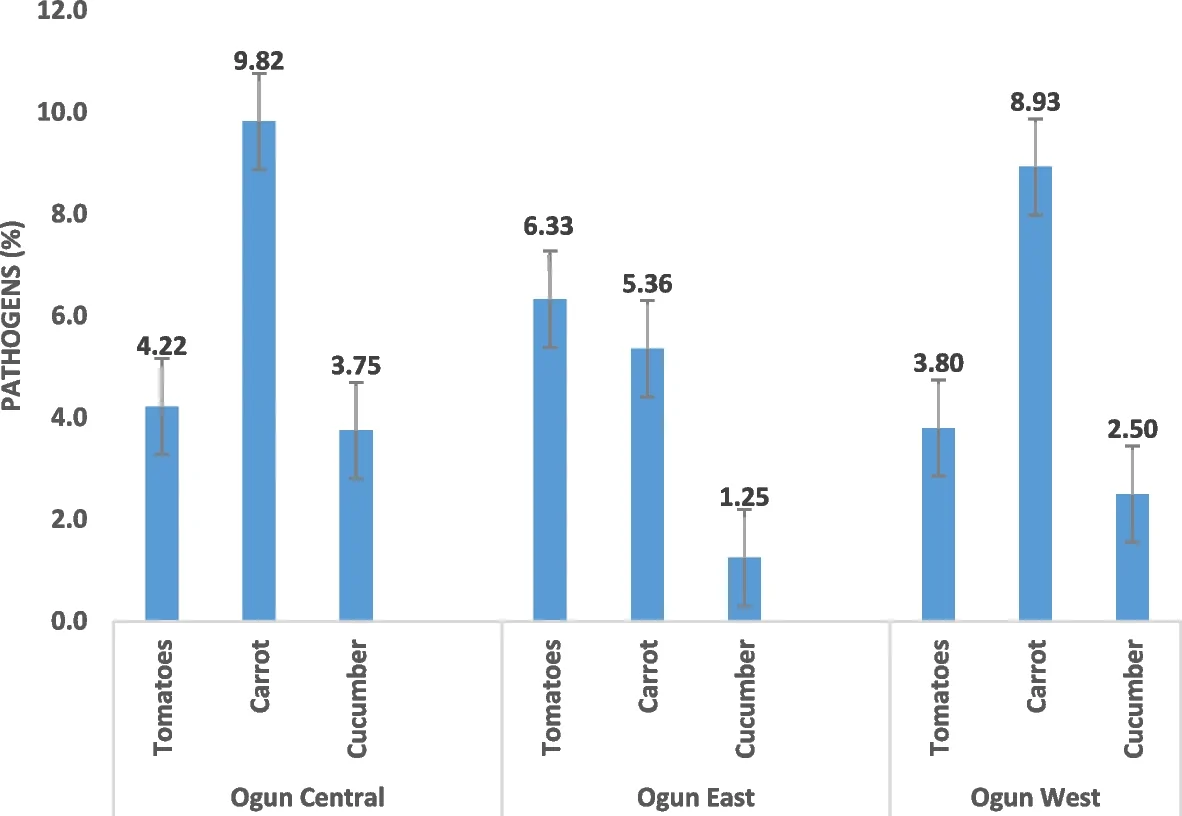
Overall prevalence of *E. coli* across senatorial districts

The overall prevalence of *E. coli* in the selected vegetable samples in all the 3 senatorial districts is presented in Fig. 2. The chart also presents the contamination rate of *E. coli* from vegetable samples collected during the 3 sampling periods. In Ogun Central, *E. coli* contamination in carrots was 9.8% (11/112), while 8.9% (10/112) was observed in carrots from Ogun West. In Ogun East, contamination in cucumber samples was 1.3% (2/160). These values describe the distribution of contamination across vegetable types and districts during the study period.

### Seasonal distribution of *E. coli* – dry season

**Fig. 3:**
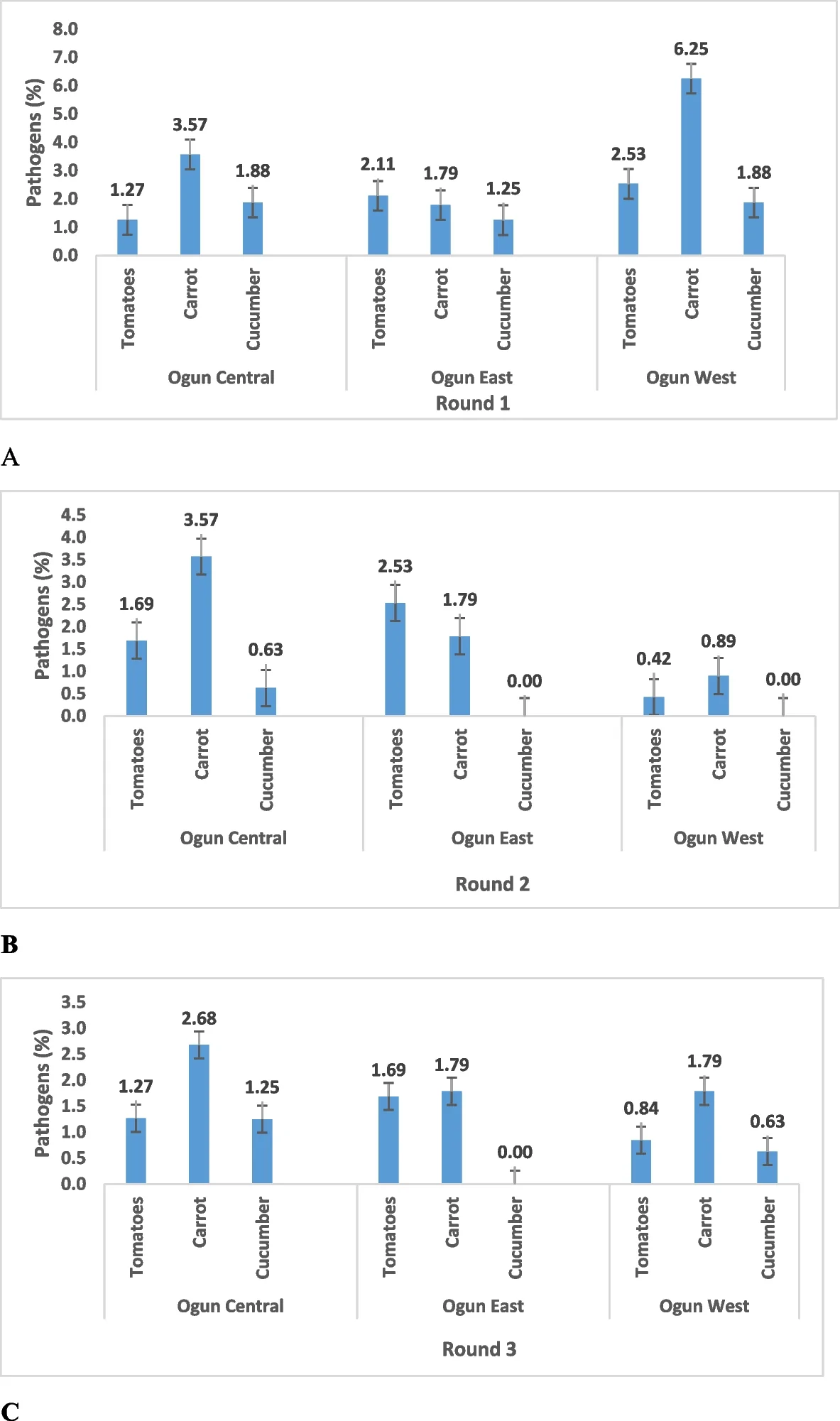
Seasonal distribution of *E. coli*. **A** Round 1 (Dry season), **B** Round 2 (Early rainy season), **C** Round 3 (Late rainy season)

The seasonal distribution of *E. coli* in the selected vegetable in all 3 senatorial districts was determined at the first round of sampling in March (dry season) and presented in Fig. 3a. The observed *E. coli* contamination rate was 6.3% (7/112) in carrot samples from Ogun West. Contamination rates of 1.3% (2/160) were recorded in cucumber samples from Ogun East and 1.3% (3/237) for tomato samples from Ogun Central.

### Seasonal distribution of *E. coli* – early rainy season

The seasonal distribution of *E. coli* in the selected vegetable in all 3 senatorial districts was determined at the second round of sampling in May (Early rainy season) and presented in Fig. 3b. *E. coli* contamination was 3.6% (4/112) in carrots from Ogun Central and 0.4% (1/237) in tomatoes from Ogun West.

### Seasonal distribution of *E. coli* – late rainy season

The seasonal distribution of *E. coli* in the selected vegetable in all 3 senatorial districts was determined at the third round of sampling in October (Late rainy season) and presented in Fig. 3c. *E. coli* contamination was 2.7% (3/112) in carrots from Ogun Central and 0.6% (1/160) in cucumbers from Ogun West.

### Number of *E. coli* isolates recovered from the selected vegetables in all the senatorial districts

A total number of 34 isolates after molecular confirmation were recovered from the selected vegetable samples collected in all 3 rounds and from all senatorial districts, including 12 from Ogun Central, 9 from Ogun East, and 13 from Ogun West (Table 3).

**Table 3:** Number of *E. coli* isolates recovered from the selected vegetables in all the senatorial districts

Senatorial districts	Number of isolates
Ogun Central	12
Ogun East	9
Ogun West	13
Total	34

### Antimicrobial susceptibility profile of isolates from vegetables in all the senatorial districts

**Fig. 4:**
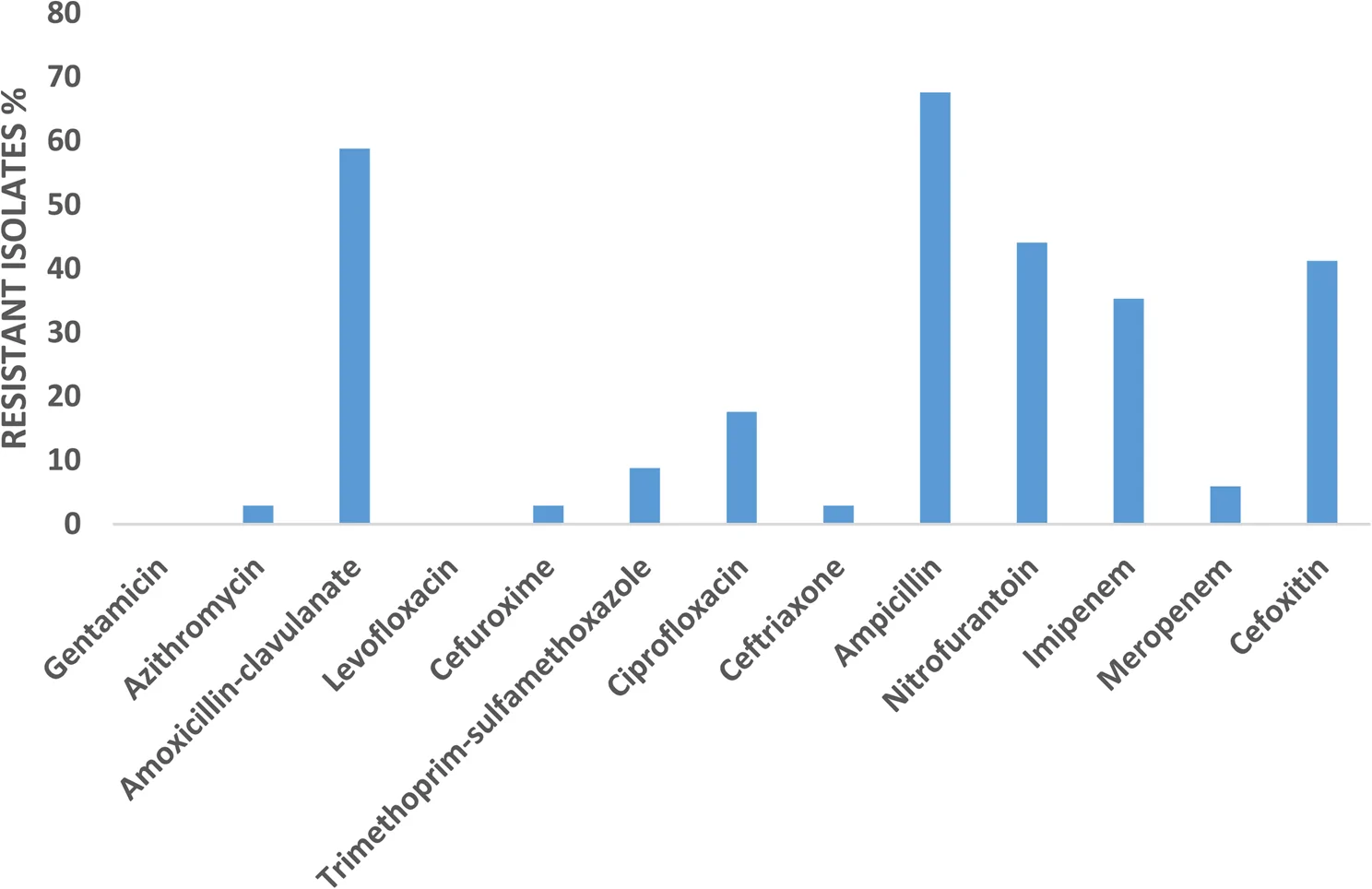
Antimicrobial susceptibility profile of isolates from vegetables in all the senatorial districts

The antimicrobial susceptibility test results for the isolates recovered from the vegetables are presented in Fig. 4. The isolates were resistant to all antibiotics except gentamicin and levofloxacin. Very low resistance was found to azithromycin (2.9%), cefuroxime (2.9%), trimethoprim-sulfamethoxazole (8.8%), ciprofloxacin (17.6%), ceftriaxone (2.9%), nitrofurantoin (44.1%), imipenem (35.3%), meropenem (5.9%), and cefoxitin (41.2%). Furthermore, the Chi-square analysis (Table 4) revealed a highly significant difference in resistance frequencies among the tested antibiotics (χ^2^ = 73.152, p < 0.001), indicating that the distribution of antibiotic resistance was significantly associated with the type of antibiotic tested.

**Table 4:** Chi-square analysis of antibiotic resistance frequencies among *E. coli* isolates

Parameters	Values
Chi-square	73.152
Degree of freedom	9
*p*-value	< 0.001

### Number of *E. coli* isolates possessing virulence genes and resistance genes in the selected vegetable from all the senatorial districts

**Fig. 5:**
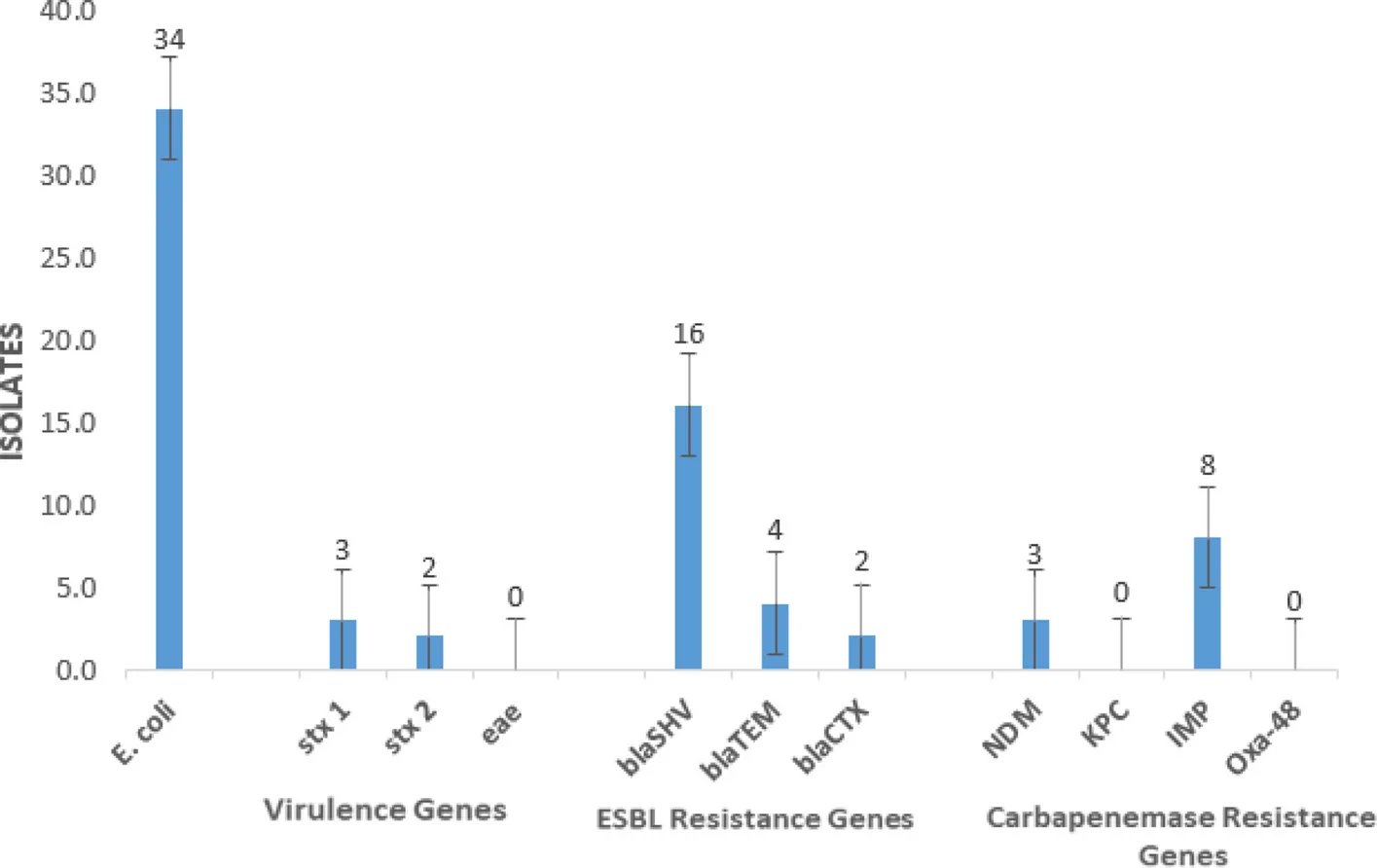
Number of *E. coli* isolates possessing virulence genes and resistance genes in the selected vegetable from all the senatorial districts

Figure 5 shows the number of *E. coli* isolates that possess virulence genes and resistance genes in the selected vegetables obtained from all the 3 senatorial districts. Out of 34 isolates recovered from *E. coli*, *stx1* was detected in 3 isolates, *stx2* in 2 isolates, and *eae* was absent in all isolates for the virulence genes. For the Extended-spectrum Beta-lactamase (ESBL) resistance genes, *blaSHV* was found in 16 isolates, *blaTEM* in 4 isolates, and *blaCTX* was detected in 2 isolates. Carbapenemase resistance genes showed that *NDM* was present in 3 isolates, *IMP* in 8 isolates, while *KPC* and *Oxa-48* were absent in all isolates.

## Discussion

Fresh produce, commonly consumed in its raw or minimally processed form, presents a greater risk for transmitting foodborne pathogens and AMR compared to other food types (Kläui et al. 2024). Despite the nutritional richness of vegetables, they have been pinpointed as carriers of pathogenic microorganisms resistant to antimicrobials, which poses significant concerns for food safety, particularly given their frequent consumption in their fresh state (Olanbiwoninu et al. 2024). This study identified a total of 34 *E. coli* isolates from the vegetable samples, including 12 isolates from Ogun Central, 9 from Ogun East, and 13 from Ogun West. Overall, *E. coli* contamination was low across all vegetable samples in the 3 senatorial districts. The *E. coli* isolates demonstrated variable resistance patterns. No resistance was detected against gentamicin and levofloxacin. The high level of antibiotic-resistant bacteria found in the vegetable samples is concerning and suggests that vegetables may play a role in the spread of antibiotic-resistant bacteria. For example, antibiotics are regularly used in livestock, poultry, and fisheries, and their waste is often used as manure for crops. Additionally, farm waste and effluents can contaminate water bodies, which may then be used for irrigation, leading to the spread of antibiotic resistance in raw vegetables (Odewale et al. 2023).

More so, the significant variation in antibiotic resistance patterns among the tested antibiotics can be attributed to differences in usage, mechanisms of action, and the adaptive capacity of *Escherichia coli*. High resistance levels are commonly associated with widely used antibiotics, particularly β-lactams such as ampicillin and amoxicillin-clavulanate, where prolonged exposure promotes the selection of resistant strains through mechanisms including β-lactamase production, modification of penicillin-binding proteins, and efflux pump activity (Nasrollahian et al. 2024). In contrast, antibiotics with low or no resistance are likely to be used less frequently or more strictly regulated, resulting in reduced selective pressure. Environmental factors further contribute to this variability, as antibiotic residues in soil and water can enhance the persistence and spread of resistance genes via horizontal gene transfer mechanisms such as plasmids, transposons, and integrons (Nasrollahian et al. 2024).

This finding aligns with previous reports that have identified antibiotic-resistant bacteria in fruits and vegetables. For instance, Olanbiwoninu et al. (2024) reported the presence of antimicrobial-resistant pathogens in fruits and vegetables from retail and home gardens in Oyo, Ogun, and Ekiti States in southwestern Nigeria. Their study found high resistance to cefuroxime, ceftriaxone, and amoxicillin-clavulanate, which contrasts with the present study. However, the low resistance to ciprofloxacin and gentamicin reported by Olanbiwoninu et al. (2024) is consistent with the findings of this study. While some resistance patterns observed in this study align with previous reports, discrepancies were also noted. These differences may reflect variations in sampling strategies, types of vegetables analyzed, the panel of antimicrobials tested, and the microbiological methods used for isolation and identification. Oyedele et al. (2020) evaluated the bacteriological safety of pineapples and watermelons from urban markets in six south-western Nigerian states, finding co-resistance to ampicillin and gentamicin.

Vegetable contamination can occur at various stages of the value chain, from the farm (through sources such as soil, seeds, manure, agrochemicals, farm equipment, and irrigation water) to the retailer, where factors like handler hygiene and storage conditions play a role (Somorin et al. 2023). The presence of bacterial pathogens like *E. coli* in vegetables sold by market traders may be due to transmission from irrigation water, manure, soil, wash water, or even the hands of traders or customers. Al-Kharousi et al. (2019) also reported the presence of ESBL-producing *E. coli*, which is consistent with the findings of this study. Some of the isolates recovered from the vegetables in this present study exhibited low resistance to amoxicillin-clavulanate, which belongs to the ESBL group. This may be due to possibly due to low gene expression, silent genes, or limitations in the sensitivity of the phenotypic testing methods (Stasiak et al. 2021).

This study identified the presence of virulence and resistance genes in the isolates recovered from vegetables. The detection of these virulence factors highlights the significant public health risk posed by the contamination of vegetables with *E. coli*. While virulence genes are common in pathogenic bacteria, the study found a low overall presence of these genes among the recovered isolates, and no single isolate tested positive for all the virulence genes examined. Furthermore, the discovery of unique virulence patterns among different pathogens illustrates the complexity of potential infections and emphasizes the need for thorough surveillance and control measures to reduce the public health risks associated with these pathogens (Duma et al. 2024). Comparing these results with those from the disk diffusion test revealed inconsistencies between the presence of resistance genes and the actual phenotypic expression of antibiotic resistance in the tested samples.

The study found a low prevalence of *stx1* and *stx2* genes and an absence of the *eae* virulence gene in *E. coli* isolates. This result aligns with Jongman and Korsten (2017), who also reported the absence of the *eae* gene in *E. coli* isolates from various leafy greens. The presence of *stx2* in certain *E. coli* isolates indicates potential reservoirs of virulence genes, with the possibility of emerging pathogenic *E. coli* strains (Olaniran et al. 2009). The detection of the enterohemorrhagic *stx2* gene suggests that these *E. coli* isolates may have the potential to cause Shiga toxin–related diseases (Aijuka et al. 2014). However, *E. coli* strains carrying *stx2* without the *eae* gene are not typically associated with foodborne disease outbreaks, although some genetic elements may be mobile, potentially creating new virulent strains (Käppeli et al. 2011). The ability to produce Shiga toxins, such as *stx1* and *stx2*, is a key virulence factor of *E. coli* (Melton-Celsa 2014). STEC strains can cause a range of infections, from mild, often unnoticed diarrhea to more severe conditions like hemorrhagic colitis and the life-threatening hemolytic uremic syndrome (HUS) (Guth et al. 2010).

Furthermore, *stx2* has been shown to be more virulent compared to *stx1* alone or a combination of *stx1* and *stx2* (Tahamtan et al. 2010). Numerous studies have reported the presence of the *eae* gene in *E. coli* isolates from vegetables worldwide (Mazaheri et al. 2014; Waturangi et al. 2019; Ratshilingano et al. 2022). The *eae* gene is essential for the close attachment of the bacteria to epithelial cells, enabling *E. coli* strains with this gene to attach intimately and cause microvillus effacement in vitro and in animals (Makhado et al. 2022). In South Africa, a study evaluated the presence of virulence factor genes (*stx1*, *stx2*, *eae*, and *ehxA*) in pathogenic STEC serogroups (O157, O26, O111, O145, and O121) isolated from swine faecal samples in the Eastern Cape. Only the *stx2* gene was detected in 1.4% of the 22 *E. coli* isolates, while *stx1*, *ehxA,* and *eae* were not found (Iwu et al. 2016). Similarly, STEC O157 and non-O157 strains were isolated from the faeces of 106 beef calves in a feedlot in Gauteng, South Africa (Onyeka et al. 2021). The study also identified the presence of ESBL resistance genes in isolates from *E. coli*. *blaSHV* was more prevalent in *E. coli* isolates compared to *blaTEM* and *blaCTX*. These findings contradict those of Olanbiwoninu et al. (2024), who reported that *blaCTX*, found in *S. enterica*, *L. monocytogenes*, *B. cereus* and *S. aureus*, was more prevalent than *blaTEM* and *blaSHV*. The current study also differs from Richter et al. (2020), who found that *blaCTX* was more dominant, followed by *blaTEM* and *blaSHV*, in Enterobacteriaceae isolated from commercial spinach. Globally, *blaTEM*, *blaSHV*, and *blaCTX* are the primary ESBLs identified in clinical and agricultural contexts, including fruits and vegetables (Zhang et al. 2015).

Carbapenemase resistance genes were determined in isolates from *E. coli* and were found to be present in only a few *E. coli* isolates. This finding corresponds with the low phenotypic resistance observed to imipenem and the limited resistance to meropenem in the antibiotic susceptibility test. Specifically, *NDM* was found in only 3 isolates, and *IMP* in 8 isolates, while *KPC* and *OXA-48* were absent in all isolates. Carbapenems are critical antimicrobial agents for treating severe infections caused by MDR Gram-negative pathogens (Wu et al. 2019). MDR is defined as the development of resistance to at least one antimicrobial agent in three or more different antimicrobial classes (Magiorakos et al. 2012). Antibiotic-resistant bacteria (ARBs) and antibiotic resistance genes (ARGs) have become significant sources of carbapenemase-producing bacterial strains (Liu and Song 2019; Zhou et al. 2020). The global prevalence of carbapenemase-producing *E. coli* is a major concern in the context of antibiotic resistance (Harding-Crooks et al. 2023). Additionally, the emergence and worldwide spread of carbapenem-resistant Enterobacteriaceae (CRE), particularly NDM-producing strains, pose a serious challenge in healthcare settings (Wu et al. 2019). The detection of NDM in three isolates, though numerically low, is noteworthy given that carbapenemase genes are rarely reported in fresh vegetables. This could reflect contamination from irrigation water, soil, or handling practices, which are potential environmental reservoirs of NDM-producing bacteria (Furlan et al. 2024; Wu et al. 2019). Such findings highlight the importance of monitoring fresh produce for critical resistance determinants, even when phenotypic carbapenem resistance is low. *E. coli* is a major carrier of *blaNDM*, with certain lineages like ST167, ST410, and ST617, as well as common plasmids (IncX3, IncFII, and IncC), being associated with the global dissemination of *blaNDM* in *E. coli* (Wu et al. 2019).

Although the *KPC* resistance gene type is the most common enzyme causing outbreaks in many countries across Asia, Africa, North America and Europe (Codjoe and Donkor 2017; Taggar et al. 2020), its absence in this study could be attributed to the limited number of isolates recovered. Furthermore, a recent study by Furlan et al. (2024) identified carbapenemase-encoding genes such as *blaKPC*, *blaNDM*, *blaIMP*, *blaVIM*, *blaSPM* and *blaOXA-48* in *E. coli* isolates from vegetables. A study in Nigeria by Jesumirhewe et al. (2017) also reported the presence of *blaOXA-48* in clinical isolates, which contrasts with the current study due to the absence of *blaOXA-48* in the isolates examined. This is the first study to report the detection of carbapenemase resistance genes in vegetables from Ogun State, Nigeria.

## Conclusions

The findings of this study underscore the public health significance of antimicrobial-resistant *Escherichia coli* in fresh vegetables across the studied senatorial districts. Rather than merely reflecting localized contamination, the observed resistance patterns point to broader systemic challenges related to antibiotic use, environmental exposure, and hygiene practices within the food supply chain. These results highlight the need for strengthened surveillance systems integrating a One Health approach, particularly at the interface of agriculture, environment, and public health. Improved hygiene practices among vegetable handlers, stricter regulation of antibiotic use, and routine monitoring of antimicrobial resistance in fresh produce are critical to mitigating the spread of resistant bacteria. Future research should focus on source tracking of contamination pathways and evaluation of targeted interventions to reduce microbial risks in fresh produce systems. Addressing these gaps will be essential for developing effective, evidence-based strategies to combat antimicrobial resistance in low- and middle-income settings.

## Data Availability

The data underlying this article are available in the article and will be provided upon request from the corresponding author.
